# Innovative video tailoring for dietary change: final results of the *Good for you!* cluster randomized trial

**DOI:** 10.1186/s12966-015-0282-5

**Published:** 2015-10-07

**Authors:** Kim M. Gans, Patricia Markham Risica, Akilah Dulin-Keita, Jennifer Mello, Mahin Dawood, Leslie O. Strolla, Ofer Harel

**Affiliations:** Institute for Community Health Promotion, Brown University School of Public Health, Providence, Rhode Island 02912 USA; Department of Human Development and Family Studies and the Center for Health Interventions and Prevention, University of Connecticut, 348 Mansfield Road, Unit 1058, Room 330, Storrs Connecticut, 06269 USA; Department of Statistics, University of Connecticut, 215 Glenbrook Road Unit 4120, Storrs CT, 06269 USA

**Keywords:** Diet, Nutrition, Nutrition education, Tailoring, Fruit and vegetable, Fat, Video

## Abstract

**Background:**

Effective, low-cost approaches are needed to enhance dietary behavior change. While both video and tailoring technology have been effective interventions to improve diet, these approaches have never been combined to study the effectiveness of tailored videos. The purpose of this paper is to discuss the results of *Good For You!,* a randomized trial that tested the efficacy of innovative, individually tailored videos in helping worksite employees decrease dietary fat and increase fruit and vegetable (F&V) intake.

**Methods:**

Worksites were matched on approximate size, type of company and workforce composition and randomized to one of three experimental conditions: Non-Tailored written information (NT) (*n* = 14), Tailored Written information (TW) (*n* = 14), or Tailored Written + Tailored Video (TW + TV) (*n* = 15). Evaluation was conducted at baseline, 4 and 7 months. We used the NCI Fat Screener and an adapted Food Habits Questionnaire (FHQ) to estimate fat intake and fat-related behaviors, the NCI F&V Screener and F&V Habits Questionnaire (FVHQ) to measure F&V intake and behaviors. Generalized linear models were examined for all outcome measurements.

**Results:**

2525 worksite employees were recruited. At 4 months, dietary fat intake decreased significantly more for TW (−2.95 %) and TW + TV (−3.14 %) compared with NT (−2.42 %). FHQ scores decreased significantly more for TW + TV than the other two groups. Fruit intake increased the most for TW + TV compared to NT and TW. Both TW (1.30 cups) and TW + TV (1.59 cups) increased F&V intake significantly more than NT (0.78 cups). TW + TV showed the largest increase in F&V behaviors on the FVFQ. At 8 months, dietary fat change continued to be significantly better for TW + TV (−3.48 %) than NT (3.01 %). F&V intake increased significantly more for the TW + TV group (1.38 cups) compared to the NT group (1.04 cups) and FVHQ changes were significantly greater in TW + TV and TW than for NT.

**Conclusions:**

The tailored intervention participants were more likely to decrease fat and increase F&V intake. The TW + TV group was generally the stronger of the two tailored interventions, especially at the longer term follow-up, demonstrating the promise of tailored video as an intervention to change eating habits. Future studies should explore newer channels and technologies in addition to DVDs for delivering tailored video interventions such as the internet and smart phones.

**Trial Registration:**

ClinicalTrials.gov identifier: NCT00301678

## Background

Increasing fruit and vegetable consumption and limiting intake of dietary fat, especially saturated fat, have been recommendations of the Dietary Guidelines for Americans, the American Heart Association, and the American Cancer Society [1–3]. However, many Americans do not comply with these recommendations [4]. Effective, low-cost and innovative dietary change approaches that can reach large and diverse segments of the population are essential in order to positively impact behavior change and advance disease prevention efforts.

One-on-one dietary counseling can be effective, [5] but is too expensive for a wide-reaching public health approach. Generic self-help nutrition education materials are lower in cost and can reach more people, but may not be as effective at the individual level [6]. The challenge is to create materials and interventions that combine the effectiveness of interpersonal communication with the efficiency of mass-mediated communication.

Using computer programming to tailor education materials to an individual’s needs and interests is a potential solution as it mimics the process of interpersonal interaction and is much less expensive to deliver than one-on-one counseling. Tailoring is defined as any combination of information or change strategies intended to reach one specific person, based on characteristics that are unique to that person, related to the outcome of interest, and have been derived from an individual assessment [7, 8]. There is a growing body of evidence suggesting that tailoring educational materials is more effective in producing dietary and other healthy behavior changes than traditional non-tailored, self-help approaches [6, 9–14].

Video is another potential effective educational method for enhancing behavior change [15–18]. Video has advantages over other media as it can be watched with other household members, information can be standardized, viewing can be repeated, and video lends itself to explaining concepts that are difficult to communicate in print [17, 18]. Further, with the virtual ubiquity of audiovisual equipment in U.S. households, video is a format that can reach almost all Americans. Almost all (98 %) of U.S. homes have a television, [19, 20] and television reaches more adults each day than any other medium across all demographic groups [21]. Most homes (85 %) own at least one DVD player and many watch DVDs on computer or internet, especially ethnic minorities [20, 22–25]. Newer options include streaming video and video-sharing websites, which can be accessed via computer or smart phone.

While both video and tailoring technology have been used to improve dietary changes, no studies have yet combined these approaches to test the effectiveness of tailored videos on changing health and/or dietary behavior. The purpose of this paper is to discuss the results of a randomized trial entitled *Good For You!* that tested the efficacy of individually tailored videos in helping individuals decrease dietary fat and increase fruit and vegetable intake.

## Methods

*Good For You!* was funded by the National Cancer Institute (Grant # CA86066). The aims of this project were 1. To develop an innovative intervention (tailored take-home videos/DVDs with accompanying tailored written materials) to help individuals improve their eating habits (decrease fat and increase F&V intake); and 2. To conduct a randomized controlled trial to test the effectiveness of this approach in achieving these dietary changes as compared to written tailored materials only or non-tailored materials. Study protocols for the research were approved by the Brown University Institutional Review Board.

We chose to conduct a randomized trial in worksites because we envisioned that worksites could be a potential future site for dissemination of the intervention delivered through organizations such as third party payers that offer wellness programs to worksites. Blue Cross Blue Shield of RI (BCBSRI) was interested in participating in the study and potentially replicating/disseminating the *Good For You!* intervention as part of their worksite wellness programs if it was found to be effective. We created an Advisory Board including representatives from BCBSRI, a local hospital worksite wellness program, the RI Department of Health and the CEO of a large worksite in RI. The purpose of the Advisory Board was help us to recruit sites, plan the intervention and evaluation methodologies and strategize around problems.

The first phase of the study was formative research to inform the development of the intervention. A mixed-methods triangulation approach including qualitative exploratory focus groups, a quantitative telephone survey, and qualitative confirmatory focus groups was conducted with employees from various worksites. Exploratory focus groups (*n* = 6 groups with 46 total participants) were conducted at three different worksites (ranging in size from 250–350 employees) to gather information about barriers, facilitators, and motivators for healthy eating and to determine important factors affecting food choice and behaviors. This information was incorporated into the content of a phone survey (*n* = 216 employees from five worksites) to enumerate and narrow the foci in terms of eating habits, barriers, facilitators and motivators of dietary change that would be considered for message development. Lastly, five confirmatory focus groups were conducted with 41 employees from five different worksites (ranging in size from 110–2000 employees) to confirm and clarify general conclusions drawn from the exploratory focus groups and telephone survey, to explore the acceptability of behavioral strategies and study logistics and to pre-test scenarios and messages for intervention materials. Results from the formative research determined the content of the tailored written and video interventions and the format of the video segments.

### Worksite recruitment

To identify potential worksites, we obtained company names through Reference USA lists, the RI Department of Economics list of largest employers in RI, and lists of companies participating in the Worksite Wellness Council of Rhode Island. Worksites were eligible if they: had at least 140 employees and were not currently conducting extensive nutrition education programming for their employees. Our original plan was to recruit 12 companies per experimental group for a total of 36 companies. Because of slow recruitment in some sites and because of the need to find appropriate matches, we recruited a total of 52 potential sites (22 by BCBSRI, 22 through calling without prior contact and 8 through advisory board contacts). Company size ranged from 145 to 2333 employees and were from New England, the Midwest and the Southern U.S. Companies were selected in groups of 3 matched on approximate size, type (e.g. manufacturing; information; and professional, scientific, technical), and workforce composition (e.g. blue/pink/white collar mix, number of salary v. hourly employees). Triplets of worksites began participant recruitment simultaneously with one triplet starting approximately every 2 months. Five of the 52 initially recruited worksites were ultimately not able to participate before matching and baselines began. In addition, several smaller worksites with low initial recruitment were ultimately combined with other similar sites prior to randomization and two smaller hospital sites were combined prior to randomization so that four hospitals that were part of the same system could be rolled out together. Thus, a total of 43 worksites were ultimately involved in the study. Randomization to one of the three experimental groups occurred after baseline measurements were completed: 1. Non-Tailored written information (NT) (14 worksites); 2. Tailored Written information (TW) (14 worksites); 3. Tailored Written information + Tailored Video (TW + TV) (15 worksites).

### Employee recruitment

Participant recruitment methods differed somewhat by worksite but generally included posters, flyers, emails, an on-site kick-off event, payroll stuffers, table tents, home mailings, blurbs in newsletters and piggybacking with other events already taking place such as health fairs and employee appreciation days. Participants could contact project staff via email or toll-free telephone line. Project staff would then mail potential participants the registration/consent materials with a business reply envelope to mail the completed materials back. Participants could also fill out the registration materials in person at the on-site events.

### Randomization and intervention groups

After baseline recruitment was completed, all worksites were randomly assigned by the data manager (using a computer assigned system) to one of three intervention groups: 1. Non-Tailored written information (NT), which received three separate mailings of traditional nutrition education and other wellness brochures (see below). The nutrition brochures contained messages that were consistent with the tailored intervention. Upon study completion, NT participants had the option of receiving the tailored nutrition materials; 2. Tailored written information (TW), which received three separate mailings of written materials tailored for participants; 3. Tailored written information + tailored video (TW + TV), which received three separate tailored videotapes plus the tailored written materials. The tailored materials were mailed at three time points: one week after the baseline survey was completed, four weeks after mailing one, and four weeks after mailing two.

Tailoring was based on participants’ answers to the baseline telephone survey and two brief “re-tailoring” assessments that were conducted by mail or phone after mailing 1 and 2 to determine the information that would be included in the participant’s next packet and/or video. The baseline tailoring questions were based on the following: Dietary intake data from the screeners were used to provide micro-tailored feedback about current fat and F&V intake to participants in comparison to national guidelines. Feedback from the FHQ and FVFQ about fat and F&V-related behaviors of participants was also provided. Then participants chose food and meal pages for topics where they had a need for behavior changes. Questions were also asked about participants’ personal motivators; barriers, and other psychosocial issues related to healthy eating and participants chose pages based on their needs and interests. In addition, participants were asked to choose special topics of interest to them. See Tables 1, 2 and 3. Further detail is available from the authors.

**Table 1:** Content areas for the tailored written materials

Content area first mailing	Topics in library	Participant receives
Benefits/Motivators (Lose weight/look better, Feel better, Be Role Model, Prevent Disease)	4	2
Feedback on Fat and F&V Intake and Comparison to Guidelines	1	1
Psychosocial Topics (Self-talk, Emotional eating, Creating habits, Somatic issues, Social support, Environmental Restructuring)	6	2
Barriers (Cost, Taste, Availability, Time)	5	1
Food and Meal Ideas		
Fruit and Vegetable-related	4	1
Fat-related	14	2
Meal-related	5	1
Goal Setting and Action Plan (NT)	1	1
Special Interest Pages (Pesticides, Getting kids to eat F&V, Label Reading, Vegetarian, Cholesterol-lowering, Exercise, Diets, supplements, Glycemic Index, Herbal supplements, Constipation, Hypertension)	13	2
Recipes and Resources (NT)	1	1
**TOTAL**	54	14
**Content Area Mailings 2 and 3**	Participant Receives*	
***Reinforcement and Cognitive Restructuring***	**1 topic**	
Psychosocial Topics	Part 2 of topic received in mailing 1	
***Barriers***	**1 topic**	
Food and Meal Ideas		
Fruit and Vegetable-related	1 topic	
***Fat-related***	**2 topics**	
***Meal-related***	**1 topic**	
Goal Setting and Action Plan	1	
***Special Interest***	**2 topics**	

Goal setting and recipes & resources sections were not counted in “number of topics in library” because all participants got these sections. These sections did not have tailored content. The psychosocial section was not included in the count because this is part 2 of what was received in previous packets. There are also 4 cognitive restructuring topics in the library based on the participant’s success with goal setting. If the participant achieved their goal, information on continuing their success was mailed. If the participant was unsuccessful in achieving their goal, information was provided as to how to keep trying. Total topics in library = 56. Items in BOLD are re-tailored

**Table 2:** Content areas for the videos

	First tailored video		Second & Third tailored videos
Content area	Topics in library	Participant receives	Participant receives
Benefits	4	2	
**Reinforcement and Cognitive Restructuring**			1
Psychosocial Topics	6	2	Part 2 of topic received in mailing 1
**Barriers**	5	1	1
Food and Meal Ideas			
Fruit and Vegetable-related	4	1	**1**
**Fat-related**	14	2	**2**
**Meal-related**	5	1	1
Goal Setting and Action Plan (NT)	1	1	1
Special Interest	4	1	**1**
**TOTAL**	43	11	**8**

Items in BOLD are re-tailored

*The psychosocial and goal setting sections in “participant receives” were not included in the count because the psychosocial section is part 2 of what they received in previous packets. Goal setting was not in the count as well because all participants receive this section. (received = 7)

There are four cognitive restructuring topics in the library based on the participant’s success with goal setting. If the participant achieved their goal, information on continuing their success was mailed. If the participant was unsuccessful in achieving their goal, information was provided as to how to keep trying. Total topics in library = 46

**Table 3:** Baseline demographic characteristics (*n* = 2525)

Demographic characteristic	Category	Total % (*n*)
Gender	Female	80.7 % (2038)
Age category in yrs	18-29	16.1 % (406)
	30-39	19.3 % (487)
	40-49	30.7 % (773)
	50-59	26.4 % (664)
	60 and up	7.8 % (195)
Education	<8^th^ grade/Some HS/HS/GED	16.8 % (425)
	SomeTech/CC/Some Coll/Tech/CC Grad	33.2 % (839)
	College Grad	31.8 % (802)
	Post Graduate	17.3 % (438)
	Other	0.8 % (21)
Race	Black/AA	5.3 % (134)
	White	88.6 % (2236)
	Asian	1.9 % (48)
	American Indian/Alaskan Native	0.2 % (5)
	Other/Mix/Unknown	4.0 % (102)
Ethnicity	Hispanic	3.1 % (78)
Employment status	Full-time	83.8 % (2117)
	Part-time	14.6 % (368)
	Not Employed	1.6 % (40)
Payment type	Salary	44 % (1111)
	Hourly	54.1 % (1367)
	Piece work	0.4 % (10)
	Other/Volunteer	1.5 % (37)
Job type	Scientific/Technical	23.1 % (583)
	Professional/Managerial	30.1 % (782)
	Clerical/Office/Sales	31.7 % (799)
	Skill/Craft	8.3 % (209)
	Service worker	1.5 % (39)
	Manual labor	2.5 % (62)
	Machine operator	0.8 % (19)
	Other/Don't know	1.0 % (26)

*HS* High School, *Tech* Technical school, *CC* Community College

The “retailoring” surveys were mailed two weeks after the tailored intervention packets were mailed. Because of the complexity of the computer programming involved, and the time required of participants, we did not reassess all questions asked at baseline, but asked a smaller subset of questions to see if participants had attempted changes, which barriers were encountered, which food categories they wanted to work on next, and specific areas on which they wanted more information. If a participant did not mail back the retailoring survey, the participant was called to complete the survey on the phone. If the re-tailoring survey was not completed, the materials were defaulted to baseline responses to prepare the next set of materials.

Participants received a *Good For You!* binder to hold their tailored materials. The binder had five sections, three of which were for the three installments of tailored materials and two sections that contained non-tailored topics such as goal setting, recipes and a list of resources. Participants received 28 tailored topics split over the three mailings out of a library of 56 potential tailored topics. They received a combination of macro-tailored (entire topic chosen for the participant based on survey answers) and micro-tailored (messages within a topic were tailored for the participant based on survey answers) content. This included micro-tailored feedback about participants’ current intake of fat and F&V (as measured on the baseline survey). Table 1 shows the content areas for the tailored written materials. Further detail is available from the authors. The written materials were tested for readability and were found to be at 6th grade level as measured by the Flesch-Kincaid score.

In addition to the tailored written materials, participants in the TW + TV group received three tailored DVDs or video-tapes that each contained approximately one hour of content. In the three videos, participants received a total of 24 video segments out of a possible 46 segments in the video library. Video segments mirrored the written materials. The food and meal segments were filmed in a variety of locations, with most filmed in a kitchen with a chef giving cooking demonstrations, augmented by food and meal segments filmed at worksite, restaurant, grocery store and home locations. The videos also featured a variety of other segment styles such as testimonials from real people that had succeeded in making changes, vignettes telling stories, and educational segments featuring experts in the field including registered dietitians, a psychologist and a chef. The videos focused mostly on real people modeling positive behaviors and/or providing narratives about behavior change, which has been shown to be more effective than using only spoken or graphically represented health information [17, 18]. Videos also focused on gain-framed (emphasizing the advantages of a dietary behavior changes) rather than loss-framed (highlighting the negative aspects of non-compliance), which has been shown in the literature to be more empowering [18, 26, 27].

**Fig. 1:**
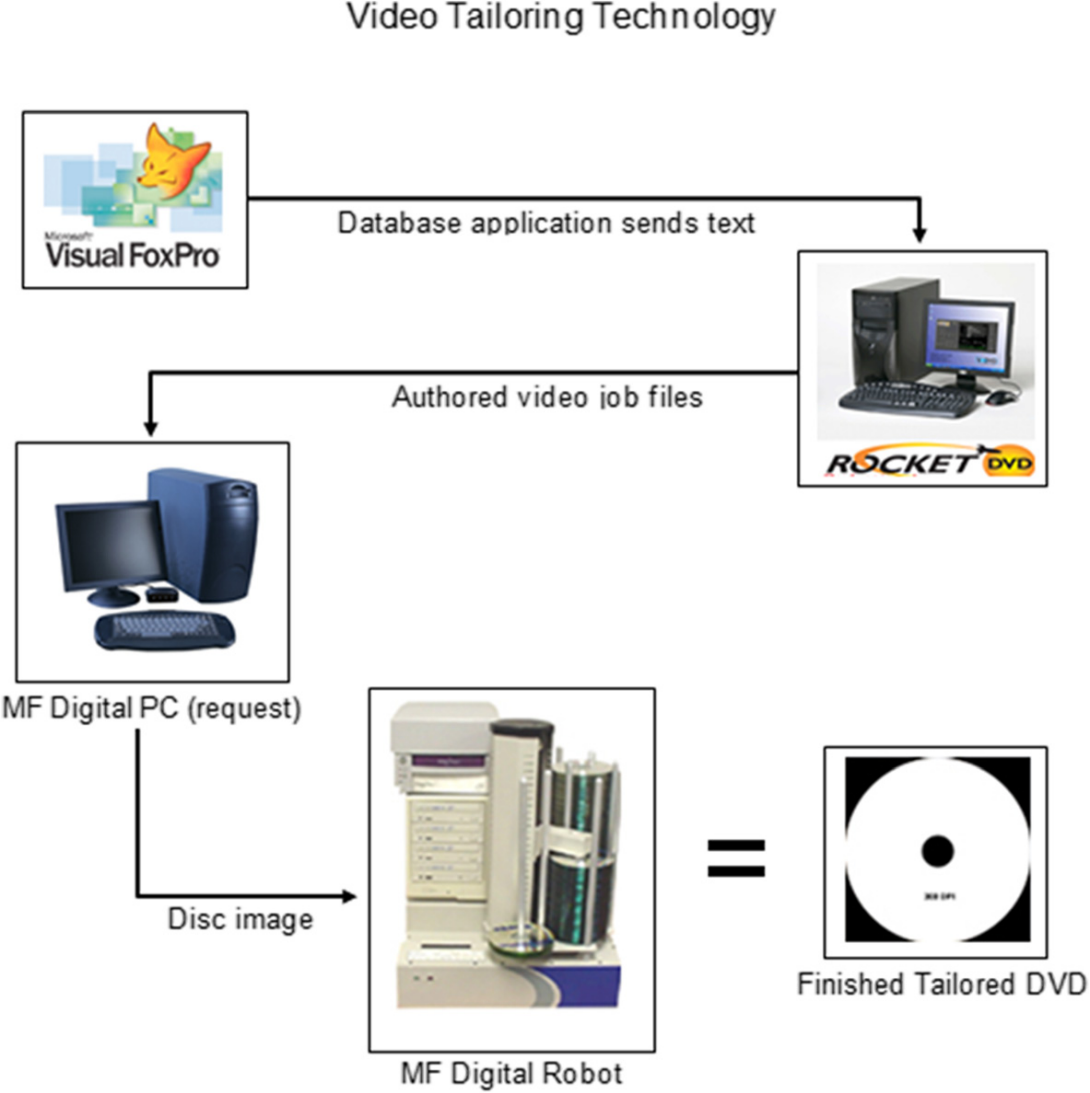
Video Tailoring Technology

To create each tailored video, participants’ telephone survey responses were entered into an in-house developed software application which collected all data using a FoxPro® database (see Fig. 1). The FoxPro® application created a tailored text file for each participant based on algorithms that chose specific segments (video and audio) depending on the participant’s survey responses. This file was used to create a job file for a Rocket DVD® application, which then processed the file, “mixed” the video and created a disc image. This image was sent to the MF Digital® PC that processed it and sent the image to the duplicator tower, which burned a blank DVD with the participant’s video, printed a tailored label with the participant’s name and dispensed onto a spindle where it was picked up by project staff to mail.

The NT group received nationally available materials purchased from national health promotion agencies such as Wellness Council of America, American Cancer Society, etc. Each of the three mailings in the NT group included a nutrition brochure with content that was very similar to the content in the TW and TW + TV groups (Guide to Eating Healthy and Being Active; Eating Well With No Time & No Money; Cooking Smart; and The New American Plate - A fresh way of looking at what you eat every day). Each mailing also included one non-nutrition-related brochure (Coping with Stress; Family Health History, and Managing Your Time).

### Evaluation measures

Evaluation measures were collected at 3 time points: baseline, 4 and 8 months. In addition to the evaluation questions, the baseline survey also included questions for tailoring purposes and the follow-up surveys included process evaluation questions.

### Fat intake

Percentage of calories from fat was estimated using the validated NCI Fat Screener [28]. To measure fat-related behaviors, we used the Food Habits Questionnaire (FHQ), [29–33] which asks a series of questions about frequency of certain foods and then follow-up behavioral questions about these foods related to fat content. For example, the FHQ asks “How often did you eat chicken in the past month” (very often, often, sometimes, rarely or never)? Then if the answer is anything other than “never”, behavioral questions are asked such as “How often do you remove the skin from chicken before eating?” (always or almost always, often, sometimes, rarely or never). Eleven behavioral questions were reverse scored so that the higher fat food behavior = 5 and lower fat = 1. Final scores were then calculated as the product of the introductory item score (4 for always, 0 for never) by the behavioral item (5 for very often, 1 for never) and multiplied by 0.25 for a final range of values for each behavioral item product of 0 to 5. This scoring system was slightly adapted from the original FHQ, which used yes or no questions for the introduction food frequency questions and only calculated an FHQ score from the non-missing behavioral questions. We have found that quantifying both the frequency of the food and the related behavioral questions provides a better picture of the respondents eating pattern and is more responsive to change. A calibration sub-study was conducted to compare the baseline FHQ score with baseline dietary fat measures from the validated NCI Fat Screener. The correlation of FHQ score with percent calories from fat on the fat screener was 0.46 (*p* < .0001), which demonstrated that the FHQ score with the adapted scoring does reflect dietary fat intake.

### Fruit and vegetable intake

F&V intake was measured using the 7-item validated National Cancer Institute (NCI) F&V screener assessment tool [34]. A Fruit and Vegetable Habits Questionnaire (FVHQ) was also adapted based on food habits questions related to F&V intake [29]. The GFY FVHQ slightly expanded the list of questions about F&V behaviors. Introductory questions were asked about how often breakfast, lunch, dinner, snacks and dessert were eaten and then F&V behavioral questions were asked about each of these meal categories. Examples include: how often was fruit eaten at breakfast, how often were vegetables added to breakfast dishes like eggs, how often were raw vegetables eaten for snacks, how often was a salad eaten at lunch?. All questions had five levels of response (always, often, sometimes, rarely or never). The responses to the introductory questions were multiplied by the responses to corresponding behavioral questions. The mean of all the products was taken to get the FVHQ. All items were scored so that higher scores are indicative of higher fruit and vegetable consumption behaviors.

## Statistical methods

All statistical and test procedures were performed with SAS software version 9.4 (SAS Institute, Cary, NC). Descriptive statistics were obtained with mean and standard deviation for continuous variables and frequencies and proportions for categorical variables. Mean change scores were calculated as the outcome (month 4 or 8) minus the baseline value. To decrease the potential for bias associated with missing values, missing values were imputed for all variables as the baseline value plus the mean change reported by those with follow-up data in the control group. Prior to this, we ran several different imputation procedures and found no differences in the results. We hence decided that the methods are robust to the imputation procedure. Descriptive statistics were computed for each outcome variable by intervention groups (NT vs. TW vs. TW + TV).). Linear models were constructed for all change in outcome measurements entered as the dependent variable with independent variables of worksite entered into the class statement (to adjust for intraclass correlation among worksites), baseline of the outcome, gender, job code (to adjust for baseline differences between groups), and group. Robust standard errors are presented in the tables. P values for the individual experimental group differences were calculated.

## Results

### Employee characteristics

**Fig. 2:**
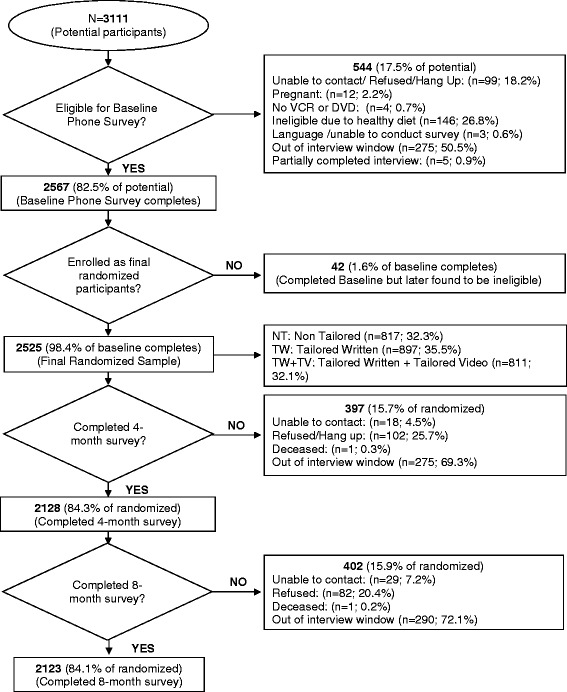
*Good For You!* Study Participation Flow Chart

A participation flow chart is shown in Fig. 2. Overall, 2525 worksite employees were randomized. See Table 4. The study population was mainly female (80.7 %) and the majority were aged 40–59 (57 %), white (89 %) and non-Hispanic (97 %). About one third had some college or technical school, while 32 % were college graduates. The majority (84 %) were employed full-time, with 54 % having white collar jobs and 44 % being paid hourly. Participants were similar across experimental groups in age, race, ethnicity and employment status, but differed by gender and job type. These characteristics were included in multivariate models to assure that group comparisons were controlled for these differences. There were no baseline differences in outcome variables by experimental group.

**Table 4:** Dietary changes in fat, fruit and vegetables measured at 4 month follow-up

Outcome Measurement	Baseline to 4 Month (mean change)											
	*n*	NT Mean change	SE	*n*	TW Mean change	SE	*p*-value (NT vs TW)	*n*	TW + TV Mean change	SE	*p*-value (NT vs TW + TV)	*p*-value (TW vs TW + TV)
SCREENER:												
(Estimated pct energy from fat)	807	−2.42	0.20	891	−2.95	0.19	0.0055*	806	−3.14	0.20	0.0004*	0.0.2598
Food Habits Questionnaire												
FHQ summary score	817	−0.36	0.02	897	−0.39	0.02	0.1138	811	−0.42	0.02	0.0004*	0.0201*
SCREENER Fruit/Vegetable:												
Fruit excluding juice	816	0.43	0.09	896	0.75	0.08	<.0001*	810	0.99	0.09	<.0001*	0.0113*
Vegetable excluding fries	817	0.36	0.16	897	0.55	0.15	0.1893	810	0.61	0.16	0.2291	0.7153
Fruit/Vegetable excluding fries	817	0.78	0.22	897	1.30	0.18	0.0037*	810	1.59	0.21	0.0002*	0.1652
Fruit & Vegetable Habits Questionnaire: (no meat)	817	2.02	0.11	897	2.26	0.09	0.0092*	811	2.65	0.11	<.0001*	0.0002*

*indicates statistical significance (*p* < 0.05)

### Process evaluation

When asked at follow-up, over 70 % of participants in the TW and TW + TV groups reported having read most or all of the tailored pages; an additional 19 % reported reading some pages and 72.6 % reported that they were still using the materials at 4-months with 54.8 % still using them at 8-months. Over 90 % thought the written materials were very or somewhat helpful and over 88 % would recommend the program to others. About 84 % of participants in TW + TV watched at least some of the first video, with over half reporting that they watched it all. About 71 and 55 % watched at least some of the second and the third video respectively, with over half reporting they watched the entire video. The mean video minutes watched for the first, second and third videos were 48, 45 and 43 min respectively. In terms of retailoring, 93.9 % of participants in the TW and TW + TV were reached for the first retailoring survey and 92.7 % were reached for the second retailoring survey.

### Participant retention

For the 4 month follow-up survey, we reached 84.3 % and for the 8 month survey, we reached 84.1 % of the baseline participants. At both time points, drop-out was not differential between experimental groups. At the 4 month follow-up, drop-outs were more likely than continuing participants to be men, younger, of lower education, mixed race and clerical staff or machine operators. At the 8 month follow-up, the drop-outs were more likely to be younger, less educated, of mixed race, hourly employed, and clerical in their job type. No differences in baseline dietary outcome variables were noted between those who continued and those who stopped participating at either 4 or 8 month time point.

### Dietary change (main outcomes)

Final outcomes are presented in Tables 4 and 5. At 4 months, dietary fat intake (as measured by the fat screener) decreased more for both TW (−2.95 %) and TW + TV (−3.14 %) groups compared with NT (−2.42 %), *p* =0.0055 for NT vs TW, and 0.0004 for NT vs. TW + TV. Similarly, FHQ scores decreased for TW + TV (−0.42) in comparison to TW (−0.39), and NT (−0.36), with TV + TW significantly different from both comparison groups (0.0004 for NT vs. TW + TV, and 0.0201 for TW v. TW) + TV). Fruit intake increased for all groups with TW + TV increasing the most (0.99 cups), TW next (0.75 cups) and NT the least (0.43 cups), (*p* < 0.0001 for NT vs TW, and for NT vs. TW + TV, and 0.0113 for TW v. TW + TV). Vegetable intake change was not statistically different by group, but there was a trend in the same direction as for fruit. F&V together increased for all groups, with both TW (1.30 cups) and TW + TV (1.59 cups) increasing more than NT (0.78 cups), *p* = 0.0037 for NT vs TW, and 0.0002 for NT vs. TW + TV); however, there was no statistically significant difference between TW + TV and TW. The FVHQ increased (meaning higher FV intake) for all groups with TW + TV showing the largest change (2.65) compared with TW (2.26) and NT (2.02), (*p* = 0.0092 for NT vs. TW, *p* < .0001 for NT vs. TW + TV, 0.0002 for TW v. TW) + TV).

**Table 5:** Dietary changes in fat, fruit and vegetables measured at 8 months after the intervention start

Outcome measurement		Baseline to 8 Month (mean change)										
	*n*	NT Mean	SE	*n*	TW Mean	SE	*p*-value (NT vs TW)	*n*	TW + TV Mean	SE	*p*-value (NT vs TW + TV)	*p*-value (TW vs TW + TV)
SCREENER:												
(Estimated pct energy from fat)	807	−3.01	0.21	891	−3.34	0.19	0.0944	806	−3.48	0.21	0.0253*	0.3962
Food Habits Questionnaire												
FHQ Product method, impute missing	817	−0.45	0.02	897	−0.44	0.02	0.7758	811	−0.46	0.02	0.4614	0.2830
SCREENER Fruit/Vegetable:												
Fruit excluding juice	817	0.48	0.09	896	0.56	0.09	0.3518	810	0.66	0.09	0.0548*	0.1991
Vegetable excluded fries	817	0.57	0.11	897	0.64	0.13	0.5382	810	0.73	0.11	0.1220	0.5075
Fruit/Vegetable excluded fries	817	1.04	0.17	897	1.19	0.18	0.3928	810	1.38	0.17	0.0443*	0.3140
Fruit & Vegetable Habits Questionnaire: (no meat)	817	1.96	0.09	897	2.17	0.09	0.0018*	811	2.34	0.09	<.0001*	0.0225*

*indicates statistical significance (*p* < 0.05)

At 8 months, some of the differences observed at 4 months were no longer evident. Dietary fat change from the screener continued to be greater for TW + TV (−3.48 %) than NT (3.01 %), *p* = 0.0253. No differences in FHQ score were observed between any groups at 8 months. Increases in fruit intake were greater for TW + TV (0.66 cups) compared with NT (0.48 cups), *p* = .0548, but no differences between other groups were found. Also, no differences in vegetable consumption were found between groups. F&V intake increased more for the TW + TV group (1.38 cups) compared to the NT group (1.04 cups), *p* = .0443, but no other differences were found. The FVHQ changes were greater in TW + TV (2.34) and TW (2.17) groups than for NT (1.96). (*p* = .0018 for NT vs. TW; *p* < .0001 for NT v. TV + TW; *p* = .0225 for TW vs. TW + TV).

## Discussion

Overall, the study results demonstrate that the *Good For You!* tailored interventions were more likely to decrease fat intake and increase F&V intake than the non-tailored intervention, and that for the most part, the TW + TV group was the stronger of the two tailored interventions, especially at the longer term follow-up. This demonstrates (as in other studies) that tailored interventions are more effective than non-tailored interventions in changing diet [11, 35].

Systematic reviews suggest that tailoring is more efficacious for improving total F&V consumption than either generic nutrition education materials or no nutrition education [35–40]. Additionally, the effects of tailoring persist over the long term (≥6 months), [11, 35] which provide support for the current study results that tailoring led to significant improvements in F&V intake at the eight month follow-up. Intervention results from other studies provide support for multiple tailored/retailored materials relative to single tailored or untailored nutrition education [37, 41] A meta-analysis of tailored nutrition interventions suggest that on average, tailoring could result in a combined total increased intake of F&V by 0.59 servings per day when compared to no intervention and 0.35 servings per day when compared to non-tailored nutrition education [35]. Our study results indicate an effect size of 0.78 cups between TW + TV and the non-tailored intervention group at 4 months, and 0.34 cups between TW + TV and the non-tailored intervention group at 8 months, which is equivalent to 1.56 and 0.68 servings - equivalent to or better than the average findings in the literature.

The current study findings indicate significant improvement in dietary fat for TW and TW + TV. However existing research findings provide mixed support for tailoring on dietary fat outcomes. While one systematic review of tailored interventions did not find significant improvements in total fat intake, [11] meta-analyses conducted by Eyles and Mhurchu, [35] suggest a significant reduction in overall fat intake of 2.45 % calories from fat among those receiving tailored nutrition education relative to control groups and 2.20 % less than participants receiving generic nutrition education. We saw smaller changes – an effect size of 0.72 and 0.53 % calories from fat for the two tailored groups at 4 months and smaller effect sizes (0.47and 0.33 % calories from fat) at 8 months. The research findings of Kroeze et al. [36] also indicate that tailored interventions do not result in significant improvements in dietary fat-related outcomes when assessed by biomarkers of blood lipids rather than self-report. These equivocal findings suggest that more research is needed to determine the efficacy of tailored nutrition materials on both subjective and objective indicators of dietary fat [11]. In addition, in light of new dietary guidelines [3] since this study began, the emphasis of future nutrition interventions should switch from a focus on reducing total fat to reducing saturated fat, trans fat and solid fats. Future studies should also focus on improving other dietary factors such as increasing whole grains, reducing sodium and added sugars, and improving overall dietary quality.

This study also demonstrates the promise of tailored video as an intervention to help people change their eating habits. No other studies to date have examined the effectiveness of tailored video for improving dietary habits. A colleague did use our tailored video approach for medication adherence with older adults and found that it was feasible and well-liked by patients, [42] and several other studies are exploring video tailoring as components of web-based interventions, [26, 43] or computer touch screen interventions in physician offices, [44] but effectiveness of tailored video in dietary behavior change has not yet been reported. There have been studies of tailored internet interventions some of which have included video [45–49]. Only one of these studies focused on dietary change. Frenn et al. studied the efficacy of an eight-session Blackboard platform-delivered Internet approach with four 2- to 3-min videos delivered in seventh-grade science class and tailored to student’s stage of change [46]. The intervention was effective in reducing fat intake and improving physical activity among the students who received a higher dose of the intervention; however, the effectiveness of the videos cannot be untangled from the overall internet-based intervention.

To our knowledge, no other existing studies have paired tailored written and tailored video materials within the context of a dietary intervention study. The fact that the TW + TV intervention achieved more lasting dietary changes than TW alone may be due to the visual nature of the video intervention creating better attention to the information that led to improved retention [50]. Alternatively, it could be due to the video intervention providing better motivation for lasting change than just reading the information, or it could be that the combination of the tailored written and tailored video created a synergy where the information in the different media complemented each other and enabled participants to achieve lasting dietary changes. Another explanation for enhanced effectiveness of TW + TV compared to TW alone could be that while they both include the same content, they address different learning styles. As such, the combined intervention more comprehensively addresses the needs of different learners. While we cannot test this hypothesis in the current study, future studies could examine intervention delivery mode and learning styles to determine if this potentially explains differences in behavior change. Good For You! did not compare tailored written vs. tailored video or tailored video vs. untailored video. Future studies should examine the comparative effectiveness of these interventions and whether tailored video interventions are more effective with and without tailored written information.

Overall, the *Good For You!* intervention was delivered with high fidelity, but the viewership of the videos decreased over time with the third video being watched less than the first video. The videos were long (about 55–60 min each in total) with segments averaging 5–6 min. In the future, it might be more effective to deliver fewer videos and/or the videos could be shorter overall and with shorter segments. In addition, future studies should further examine the mechanism of change including the important mediating variables and tailored content to hone the intervention to its most crucial elements.

The current study was conducted with worksite employees who volunteered as participants and they were generally Non-Hispanic White, middle income and at least somewhat educated. Future studies should be conducted with ethnic minority, lower income and/or lower literate audiences to see if tailored video may be even more powerful with these groups. Tailoring in general has been shown to help lower educated individuals change behavior even more than higher educated individuals perhaps because the tailored materials are more personally relevant [41, 51–53]. Tailored video may be even more beneficial as it doesn’t require the ability to read and may be better at portraying difficult concepts and demonstrating cooking skills than print materials.

It is also very important to find ways to disseminate effective tailored nutrition interventions. Third party payers such as Blue Cross Blue Shield or other companies delivering worksite wellness programs are a potential distribution mechanism for tailored video and print interventions. A potential limitation for dissemination is the expense of video production and the hardware necessary for video tailoring. However, while initially video production can be somewhat expensive, the reproductions of DVDs are very inexpensive. DVDs can be reproduced for less than 70 cents each, whereas written materials with similar amounts of content cost much more, especially if in color. Thus, tailored video may be cheaper in the long run than tailored print.

Moreover, technology has changed since the *Good For You!* study began. The hardware and software that we used to create the tailored DVDs has since been simplified and is much less costly. Furthermore, at the beginning of the study, tailored DVDs made more sense than an internet intervention using video, because few individuals had internet connections that supported streaming video. With the changes in technology and the increasing penetration of computers and internet [20, 23, 54], even for ethnic minority individuals, [55] a tailored video intervention could be implemented using these channels rather than mailing tailored DVDs, reducing costs even further. Smartphones are also increasingly becoming portable televisions. The proportion of U.S. mobile phone owners who use phones to watch video is up 35.7 % since 2012 [56, 57]. Minorities are more likely to have smart phones than non-Hispanic White Americans, [58] and income appears to have less impact on mobile video and television consumption patterns [54, 59]. However, while internet and cell phone technology for video is catching up, traditional DVD players on home televisions still have more penetration in the majority of households. One of the advantages of tailored video is that it can easily be adapted for use in all three of the aforementioned media and would be worthwhile to study as a means for delivering nutrition interventions through different channels in the future as more adults continue to adopt Internet and cell phone technologies.

Enwald et al. suggest that there are three generations of computer-tailored interventions. The first generation consists of tailored print items; the second generation refers to interventions that use interactive media; and intervention via mobile devices make up the third generation [14, 57]. The current study is an example of first/s generation intervention, but the tailored video approach could be appropriate as a third generation intervention [14]. A smoking cessation intervention found that tailored video messages on the phone were more successful than tailored text or general text messages in smoking cessation especially in smokers with lower readiness to quit [60]. Several studies are also exploring the impact of video games on behavior modification using “serious video games” [43, 61, 62] and propose that tailoring in video gameplay has the potential to increase attention and subsequently increase positive behavior change [61, 63]. Further research is needed on these interventions.

Before discussing the study implications, it is important to mention several study limitations. The assessment tool that measured change in fat-related behaviors (FHQ) did not include a quantitative measure of fat intake (i.e. percent calories from fat); however, this tool has been used in other studies and was calibrated in the current study against a quantitative measure and found to reflect dietary fat intake. In addition, we had another more quantitative measure of fat intake from the fat screener. Regarding measurement of F&V intake, recent studies have shown that the NCI F&V screener may overestimate F&V intake, [33, 41, 64] but this would not have affected differences in F&V intake by group. Another limitation is that there was no measure of social desirability bias; however, it is likely that such bias would have occurred in all experimental groups. Another limitation is that the study did not include a longer term measure of dietary change (i.e. 12 months or longer). *Good For You!* originally did include a 12 month follow-up measure, but this follow-up was shortened to 8 months when funding was cut prior to the start of the study. Future studies should measure whether such tailored interventions maintain dietary changes over a longer timeframe (i.e. one year or greater). Furthermore, the study participants were mainly Non-Hispanic white and middle to upper income, which somewhat limits the generalizability of the findings, so future studies should be done with more diverse groups.

## Conclusions and implications

This study demonstrated that tailored interventions are effective methods for changing diet and that tailored video may offer even more promise. Future research should determine whether tailored video is potentially even more effective for lower income, ethnic and lower literate audiences. In addition, future studies should isolate the effectiveness of the tailored video by comparing effectiveness tailored video vs. untailored video and whether tailored video interventions are more effective with and without tailored written information. Future research should also examine the mechanisms of change. Future studies should further explore “third generation” channels for delivering tailored video interventions including the internet and smart phones in addition to DVDs for delivering tailored video interventions and develop partnerships to explore dissemination of effective interventions.

## Abbreviations

FHQ: Food Habits Questionnaire
FVHQ: Fruit and Vegetable Habits Questionnaire
F&V: Fruit and Vegetable
NT: Non-Tailored written information
TW: Tailored Written information
TW + TV: Tailored Written information + Tailored Video
NCI: National Cancer Institute
